# Relative Contributions of Spectral and Temporal Cues to Korean Phoneme Recognition

**DOI:** 10.1371/journal.pone.0131807

**Published:** 2015-07-10

**Authors:** Bong Jik Kim, Son-A Chang, Jing Yang, Seung-Ha Oh, Li Xu

**Affiliations:** 1 Department of Otorhinolaryngology, Seoul National University College of Medicine, Seoul, Korea; 2 School of Speech Language Therapy and Aural Rehabilitation, Woosong University, Daejeon, Korea; 3 Communication Sciences and Disorders, University of Central Arkansas, Conway, Arkansas, 72035, United States of America; 4 Sensory Organ Research Institute, Seoul National University Medical Research Center, Seoul, Korea; 5 Communication Sciences and Disorders, Ohio University, Athens, Ohio, 45701, United States of America; The University of Chicago, UNITED STATES

## Abstract

This study was aimed to evaluate the relative contributions of spectral and temporal information to Korean phoneme recognition and to compare them with those to English phoneme recognition. Eleven normal-hearing Korean-speaking listeners participated in the study. Korean phonemes, including 18 consonants in a /Ca/ format and 17 vowels in a /hVd/ format, were processed through a noise vocoder. The spectral information was controlled by varying the number of channels (1, 2, 3, 4, 6, 8, 12, and 16) whereas the temporal information was controlled by varying the lowpass cutoff frequency of the envelope extractor (1 to 512 Hz in octave steps). A total of 80 vocoder conditions (8 numbers of channels × 10 lowpass cutoff frequencies) were presented to listeners for phoneme recognition. While vowel recognition depended on the spectral cues predominantly, a tradeoff between the spectral and temporal information was evident for consonant recognition. The overall consonant recognition was dramatically lower than that of English consonant recognition under similar vocoder conditions. The complexity of the Korean consonant repertoire, the three-way distinction of stops in particular, hinders recognition of vocoder-processed phonemes.

## Introduction

In vocoder processing [1] or acoustic simulations of cochlear implants [2], speech signals are divided into a number of frequency bands. The temporal envelope of each frequency band is extracted and used to modulate a noise or a tone carrier (e.g., [3]). The number of frequency bands represents the number of places in the cochlea to be stimulated, thus mapping the spectral details of the synthesized signal. The temporal details of the signals are represented in the temporal envelope of each band. In cochlear implant stimulations, it is the temporal envelope that is used to modulate the amplitude of the electrical pulse trains. Both the spectral cues and the temporal cues have been shown to be important for speech perception (e.g., [2–5]).

Many studies have examined the minimal requirement of spectral information or temporal information for English speech recognition. A general consensus today is that the number of spectral channels required for speech recognition depends on the speech materials. Simple sentences in quiet conditions require only 3 to 4 channels of spectral information for recognition whereas more complex materials (in noise conditions) require 30 or more channels for an equivalent level of performance [2, 3, 5–9]. On the other hand, a lowpass cutoff of approximately 16–20 Hz for the envelope extractor is considered sufficient for speech recognition [2, 5]. Xu and colleagues (2005) examined the relative contributions of spectral versus temporal cues for speech recognition and further demonstrated a tradeoff between temporal and spectral cues for English phoneme recognition. That is, to achieve a certain level of phoneme recognition, an enhanced spectral cue could compensate for an impoverished temporal cue [5] (see [10] for a review).

The studies of relative contributions of spectral and temporal cues in English speech recognition have been extended to lexical tone recognition of Mandarin Chinese [7, 8]. The main findings of the studies are (1) that the number of spectral channels required for good tone recognition is much higher than English phoneme recognition in quiet (e.g., 30 channels) and (2) that tone recognition can benefit from a much higher lowpass cutoff frequency of the envelope extractor (e.g., up to 256 Hz), which allows a wider range of periodicity cues in the envelope. A tradeoff between the temporal and spectral cues is also evident for tone recognition [10].

Other than English and Mandarin Chinese, no studies have been done in a different language to confirm or to examine the relative contributions of spectral and temporal information for speech recognition. Korean, spoken by approximately 80 million people, is a nontonal language that is used in the Korean peninsula and in the northeast region of China. In Korean, the consonant inventory includes 22 phonemes and the vowel inventory includes 8 monophthongal phonemes and 12 diphthongs [11]. Compared to English, Korean stops are characterized by a three-way distinction of lenis, fortis, and aspirated. In terms of the vowel system, Korean vowels lack the lax-tense contrast. However, each of the 8 Korean monophthongs has a corresponding long vowel although the phonemic distinction between a short and long vowel is diminishing in younger generations. In addition, Korean has a simpler syllable structure (C)V(C) than does English.

Given that there exist such apparent differences in the phonetic inventory between Korean and English and that no studies have been done to examine the spectral and temporal cues for phoneme recognition in Korean, it is important to compare the relative contributions of spectral and temporal information in phoneme recognition of Korean to those of English. The purpose of the present study was to examine the relative contributions of spectral and temporal information to Korean phoneme recognition. The findings, if consistent with those in English, will enable us to extrapolate results from English to other languages. On the other hand, if the findings from the present study indicate discrepancies in the relative contributions of temporal and spectral information between the two languages, it will caution such extrapolations of results from English to other languages and will provide guidance in designing clinical rehabilitation strategies more specifically to Korean.

## Materials and Methods

### Subjects

Eleven normal-hearing native-Korean-speaking listeners (7 males and 4 females) ages 19 to 26 years old participated in this study. The pure-tone thresholds of each ear of the subjects were < 20 dB HL for octave frequencies between 250 and 8000 Hz. During screening with unprocessed speech materials, consonant and vowel recognition scores were > 90% correct for all subjects. The use of human subjects in the present study was reviewed and approved by the Institutional Review Board of the Seoul National University Hospital. Written informed consent was obtained from all study participants prior to the experiment.

### Stimuli

Eighteen consonants presented in a /Ca/ context each produced by one male and one female talker were included in the consonant stimulus set: ba, pa, ppa, ja, cha, jja, da, ta, tta, ga, ka, kka, ma, na, la, sa, ssa, and ha. The corresponding International Phonetic Alphabet (IPA) symbols for the consonants are /p, p^h^, p*, ʧ, ʧ^h^, ʧ*, t, t^h^, t*, k, k^h^, k*, m, n, l, s, s*, h/. Note that Korean consonant /ŋ/ is not used in initial position and the other three approximants /w, j, ɰ/ are often used in diphthongs. Therefore, those four consonants were not used in the consonant stimulus set.

Seventeen vowels presented in a /hVd/ context each produced by one male and female talker were included in the vowel stimulus set: had, hid, heod, haed, heud, hod, hud, hyeod, hyad, hwad, hyod, hwod, hwid, hyaed, huid, hwaed, and hyud. Note that the stop at the final position in /hVd/ structure is plain and unreleased. The corresponding IPA symbols for the vowels are /a, i, ʌ, ɛ, ɯ, o, u, jʌ, ja, wa, jo, wʌ, wi, jɛ, ɯi, wɛ, ju/. Because /ɥi/, /e/, and /je/ are phonetically similar to /wi/, /ɛ/, and /jɛ/, respectively, these three vowels (/ɥi, e, je/) were not included in the stimulus set. All speech tokens were recorded in a sound booth with a high-quality microphone (Neumann U87, Berlin, Germany). The sampling rate was 44,100 Hz and quantization was set at 16 bits. Individual tokens were isolated and stored in a wave format in a computer hard disk for further processing.

### Signal processing

Signal processing for acoustic simulations of cochlear implants was performed in MATLAB (MathWorks, Natick, MA) as in [5, 7]. Briefly, each speech signal was bandpass filtered (sixth-order elliptic bandpass filters). The number of bands (or channels) varied between 1 and 16 (i.e., 1, 2, 3, 4, 6, 8, 12, and 16). The overall bandwidth was 150 to 5500 Hz. The bandwidth and corner frequency of each analysis filter was determined using Greenwood’s (1990) formula (*f* = 165.4 (10^0.06x^ − 1), where *f* is frequency and *x* is the distance (mm) from the apex of the cochlea [12], based on the assumption that each band occupies an equal distance along the basilar membrane. Next, the temporal envelope of each analysis band was extracted with a half-wave rectification and lowpass filtering (second-order Butterworth with the cutoff frequency varying between 1 and 512 Hz in 1-octave steps). The temporal envelope of each band was then used to modulate a white noise that was bandpassed through the bank of analysis filters used earlier to filter the original speech signals. Finally, the modulated noise bands were added together and the resultant signals were stored in a computer hard disk for presentation.

### Procedures

The speech signals were presented inside a double-walled sound-attenuating booth through a speaker positioned 1 m from the subject at the level of the interaural axis. Stimuli were presented at 70 dB SPL calibrated with a sound-level meter (Brüel & Kjær Type 2231, Nærum, Denmark). Two graphical user interfaces (GUIs) were built in MATLAB to present the consonant and vowel tests, respectively. The GUIs displayed 18 buttons labeled with Hangul (i.e., Korean alphabet) of the 18 consonants in the /Ca/ format for the consonant test or 17 buttons labeled with Hangul of the 17 vowels in the /hVd/ format. Each subject responded by pointing the cursor to and clicking on the appropriate button in a forced-choice manner after a consonant or vowel token was presented in a random order. There were a total of 2880 tokens for consonants (i.e., 8 channel conditions × 10 lowpass cutoffs × 2 speakers × 18 consonants) and 2720 tokens for vowels (i.e., 8 channel numbers × 10 lowpass cutoffs × 2 speakers × 17 vowels). The order of the conditions (i.e., the combination of channel number and lowpass cutoff) was randomized. It took approximately five hours for each subject to complete both the consonant and vowel tests. Frequent rests were allowed during the tests. All subjects practiced for approximately an hour before the test in order to familiarize themselves with the procedure and the stimuli. During the practice session, each subject was presented with approximately 1000 randomly selected unprocessed and processed tokens and feedbacks regarding the accuracy of the responses were provided.

### Data Analysis

Data analysis was performed in MATLAB (with Statistics Toolbox) environment. Percent-correct scores of consonant and vowel recognition were first computed for each test condition. The overall recognition score for the female talker was approximately 4–5 percentage points higher than that for the male talker. In the present study, the percent-correct scores for both talkers were pooled together. The percent-correct scores were treated as binomial data. Following a logit transformation of the percent-correct data, a Generalized Linear Model (GLM) was used to examine the effects of number of channels and lowpass cutoff frequency on the consonant and vowel recognition performance [13].

## Results

### Korean phoneme recognition as a function of number of channels and lowpass cutoffs

**Fig 1:**
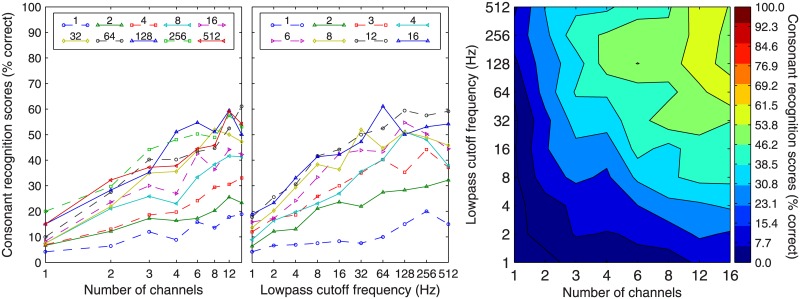
Group mean performance in Korean consonant recognition. Data represent the average of all 11 subjects. In the left panel, consonant recognition scores are plotted as a function of the number of channels, with different lines representing data for different lowpass cutoff frequencies (Hz) as indicated in the figure legend. In the middle panel, consonant recognition scores are plotted as a function of the envelope lowpass cutoff, with different lines representing data for different numbers of channels. In the right panel, contours filled with particular colors represent certain levels of performance (see color bar on the right) as a function of number of channels (abscissa) and lowpass cutoffs (ordinate).

Fig 1 shows the group mean performance in Korean consonant recognition as a function of the number of channels (left panel) and the lowpass cutoff of the envelope extractor (middle panel). The contour plot on the right illustrates the combined effects of the number of channels (abscissa) and the envelope lowpass cutoff (ordinate) on the group mean performance in consonant recognition. Korean consonant recognition depended on both the number of channels and the envelope lowpass cutoff. The GLM fitting results revealed a significant effect of the number of channels (β = .084, t = 34.1, *p* < 0.0001) and lowpass cutoff frequencies (β = .002, t = 24.2, *p* < 0.001).

The best performance for consonant recognition was approximately 60% correct, and it was achieved in conditions with ≥ 12 channels and ≥ 64 Hz of lowpass cutoff frequencies (Fig 1). This result demonstrated that a large number of channels and a high lowpass cutoff are both needed to obtain good consonant recognition scores. In addition, the contour plot (right panel, Fig 1) also demonstrated a tradeoff effect between the number of channels and the envelope lowpass cutoff on Korean consonant recognition in the less identifiable conditions. That is, equivalent consonant-recognition performance could be achieved when a smaller number of channels was coupled with a higher lowpass cutoff, and vice versa. The range of tradeoff encompassed the area covering the envelope lowpass cutoff between 1 and 64 Hz and the number of channels between 1 and 12, where the contour lines tended to run diagonally.

**Fig 2:**
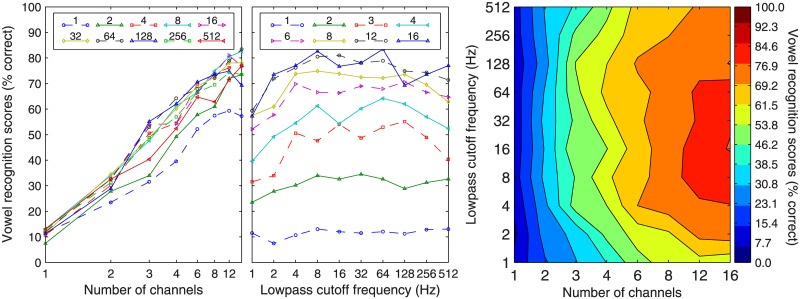
Group mean performance in Korean vowel recognition. Data represent the average of all 11 subjects. In the left panel, different lines represent data for different lowpass cutoff frequencies (Hz) as indicated in the figure legend. In the middle panel, different lines represent data for different numbers of channels. In the right panel, contours filled with particular colors represent certain levels of vowel-recognition performance (see color bar on the right) as a function of the number of channels (abscissa) and the lowpass cutoffs (ordinate).

Fig 2 shows the group mean performance in Korean vowel recognition as a function of the number of channels (left panel) and the lowpass cutoffs of the envelope extractors (middle panel). The contour plot on the right illustrates the combined effects of the number of channels (abscissa) and the envelope lowpass cutoffs (ordinate) on the group mean performance in consonant recognition. Korean vowel recognition showed great reliance on the number of channels. The dependence of the envelope lowpass cutoff was minimum. The GLM fitting results confirmed our observation. Specifically, this statistic procedure revealed a significant effect of the number of channels on the vowel recognition (β = .165, t = 57.554, *p* < 0.0001). The lowpass cutoff frequencies did not show a significant effect (β = .000, t = 0.153, *p* = 0.879).

On the right panel, it shows that the best vowel identification score was around 85% correct that was achieved with 12 and 16 channels and lowpass cutoff ranging between 4 and 64 Hz. That is, the best vowel-identification score was determined mainly by the number of channels rather than the lowpass cutoff. Further examination of the vowel identification performance in different listening conditions demonstrated that the tradeoff between the number of channels and envelope lowpass cutoff for Korean vowel recognition was not as apparent as that for Korean consonant recognition, largely due to the dominant effects of the spectral cues on vowel recognition (see Fig 2, right panel). The contours essentially showed vertically oriented lines, indicating that vowel-recognition performance was determined by the abscissa (i.e., number of channels).

### Confusion matrix analysis

**Fig 3:**
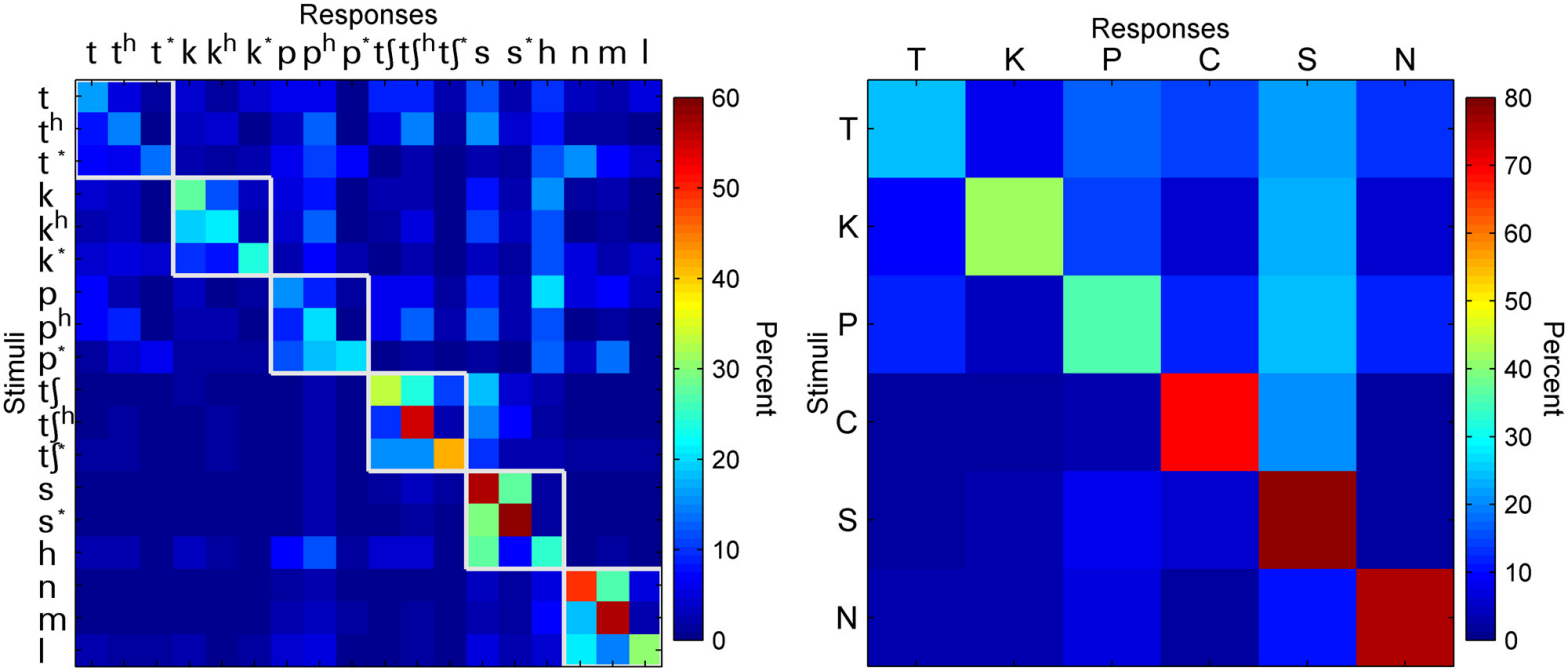
Confusion matrix of consonant recognition using data pooled across the 80 vocoder conditions (N = 11). Left panel represents confusion matrix of individual consonants. The white squares represents groups of consonants categorized on the basis of phonetic features such as the place of articulation, manner of articulation, sonorant. The y-axis represents the target consonants while the x-axis represents the recognized consonants. The value in the cell of row j and column k is the percent of times stimulus j was recognized as k. The color of each cell with reference to the color bar on the right represents the value of response percentage. Right panel represents confusion matrix of six macro categories of “T”, “P”, “K”, “C, “S”, and “N” with each of the macro categories corresponding to a white box shown in the left panel.

Fig 3 shows the confusion matrix for Korean consonant recognition. Data were pooled from all 11 subjects across all 80 vocoder conditions (i.e., 8 numbers of channels × 10 lowpass cutoffs). The color of each cell represents the percentage of responses of each stimulus that was recognized as individual Korean consonants (left panel) or individual Korean consonant category (right panel). For individual consonants, listeners had a tendency to show higher confusion among the components within each contrast for the stops and affricates that show the three-way contrast (i.e., /p, p^h^, p*/, /ʧ, ʧ^h^, ʧ*/, /t, t^h^, t*/, and /k, k^h^, k*/) (Fig 3, left panel). While for the other consonants, the confusion pattern occurred in accordance with the manner of articulation such as /s/, /s*/ and /h/ or the voicing feature for the sonorant such as /m/, /n/ and /l/. However, certain asymmetries were also found in the confusion matrix. For example, /p*/ was identified more often as /p^h^/ than /p^h^/ as /p*/. In addition, we also noticed that the responses of stop consonants were more scattered than those of the other consonants. This pattern was more evidently shown in the right panel where we divided the 18 consonants into six macro categories of “T”, “P”, “K”, “C, “S”, and “N” (each of the macro categories corresponds to a white box shown in the left panel of Fig 3) and collapsed the response data across the consonants within each macro category. Listeners tended to recognize stops as all types of consonants although the highest confusion occurred within the same place of articulation in stop consonants themselves (Fig 3, right panel).

**Fig 4:**
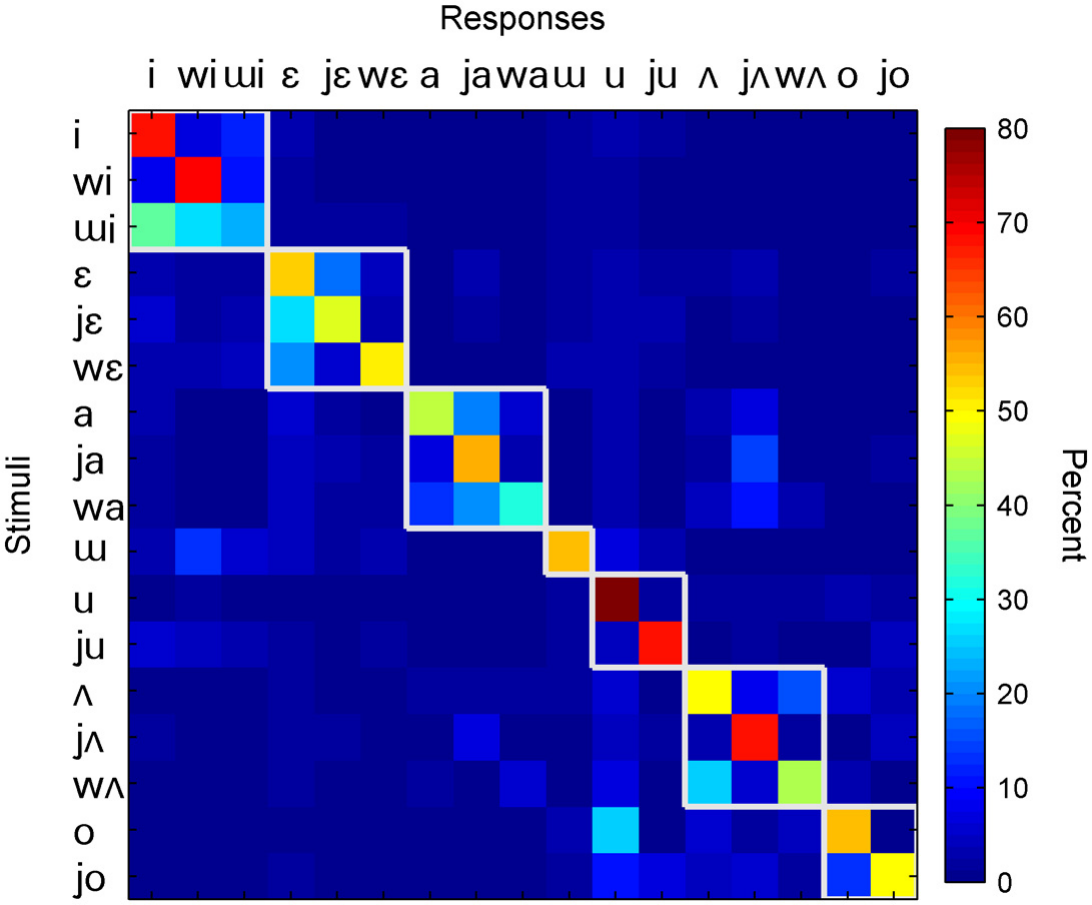
Confusion matrix of vowel recognition using data pooled across the 80 vocoder conditions (N = 11). The white squares indicate groups of vowels categorized on the basis of their nucleus. The y-axis represents the target vowel while the x-axis represents the recognized vowel. The value in the cell of row j and column k is the percent of times stimulus j was recognized as k. The color of each cell with reference to the color bar on the right represents the value of response percentage.

Fig 4 shows the confusion matrix for Korean vowel recognition. Data were pooled from all 11 subjects across all 80 vocoder conditions (i.e., 8 numbers of channels × 10 lowpass cutoffs). Higher confusion rate occurred within the groups of vowels that share the same nucleus vowel (e.g., /i, wi, ɰi/ which all contain /i/ and /a, ja, wa/ which all contain /a/) as marked by the white boxes in Fig 4. In addition, /ʌ/ and /wʌ/ showed higher confusion rate with each other than with /jʌ/. However, we also note some exceptions. In particular, /u/ and /ju/ showed little confusion between each other although they share the same vowel nucleus. On the other hand, /o/ and /u/ are two different vowels but they showed a relatively high confusion rate.

## Discussion

In the present study, we investigated the relative contributions of spectral and temporal cues to Korean phoneme recognition by systematically varying the number of channels and lowpass cutoff frequency of the envelope extractors. Our results showed that both spectral and temporal cues contribute to Korean phoneme recognition. Korean phoneme recognition improved as the number of channels increased or as the envelope bandwidth increased (Figs 1 and 2, left and middle panels). The interaction of the temporal and spectral cues on Korean phoneme recognition was manifested in the tradeoff between the number of channels and the lowpass cutoff frequency of the envelope extractor, especially for Korean consonant recognition. That is, to achieve a certain level of recognition performance, one can utilize either a large number of channels with a low lowpass cutoff frequency or a small number of channels with a high lowpass cutoff frequency (Fig 1, right panel). However, the best consonant recognition performance required both large numbers of channels and high cutoff frequencies. For vowel identification, the number of channels plays a more determinative role than the cutoff frequencies (shown in Fig 2, right panel). Such patterns as observed in Korean phoneme recognition were similar to those observed in English phoneme recognition [5].

**Fig 5:**
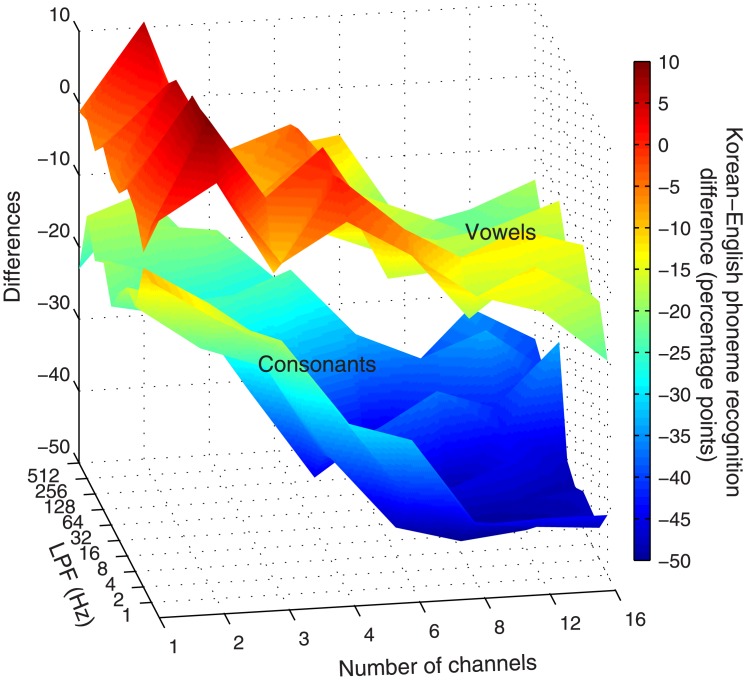
Differences in Korean and English phoneme recognition scores. The original percent-correct scores of Korean phoneme recognition scores were taken from Figs 1 and 2 of the present study whereas the original percent-correct scores of English phoneme recognition scores were taken from the Xu et al. (2005) study. Those original percent-correct scores were converted to chance-corrected scores first using the formula: P_corrected_ = (P_o_−P_c_)/(1 –P_c_), where P_o_ is the original percent-correct score and P_c_ is the chance performance. The surface plots show the differences (Korean P_corrected_−English P_corrected_) for consonants and vowels as a function of the number of channels and the lowpass cutoff frequency (LPF).

While the overall patterns of the relative contributions of spectral and temporal cues to Korean phoneme recognition bore resemblance to those of English phoneme recognition, some important differences existed. When we compare the results from the present study with those of English phoneme recognition in the Xu et al. (2005) study, the differences are remarkable, particularly in consonant recognition [5]. In both studies, we used the same 8 channel conditions and 10 lowpass cutoffs for vocoder processing. Because the numbers of English consonants and vowels were 20 and 12, respectively whereas those of Korean consonants and vowels were 18 and 17, respectively, the chance performance (P_c_) for the phoneme recognition tests was different. Therefore, to facilitate comparison between the two studies, we first converted all group mean percent-correct scores to chance-corrected scores by using the formula P_corrected_ = (P_o_−P_c_)/(1 –P_c_), where P_o_ is the original percent-correct score and P_c_ is the chance performance. The differences (Korean P_corrected_−English P_corrected_) in consonants and vowels recognition for the 80 vocoder conditions (i.e., 8 channel conditions × 10 lowpass cutoffs) are shown in surface plots in Fig 5. For almost all vocoder conditions, Korean phoneme recognition scores were poorer than English phoneme recognition scores. The mean differences of chance-corrected percent-correct scores across all the 80 vocoder conditions were 30.6 and 6.3 percentage points for consonants and vowels, respectively.

The large differences in consonant recognition performance between Korean and English are probably due to the three-way distinction of lenis, fortis, and aspirated of the Korean stops [14, 15]. For English, the voiced and voiceless contrasts of the stops are recognized with high accuracy (>75% correct) in vocoder processing with 3 or more spectral channels and ≥ 16 or 32 Hz of the lowpass cutoffs (see Fig 4 of [5]). The three-way distinction of the Korean stops might have made recognition of vocoder-processed Korean consonants particularly difficult. The acoustic differences of the three-way distinction are represented in voice-onset time (VOT), burst energy, F0, and phonation property of the following vowel, etc. [14, 16]. However, such numerous acoustic and aerodynamic characteristics of Korean stops appear to be ineffective after the vocoder processing. Although the confusion of the stops was not always confined within the three-way distinction, stop consonants /p, p^h^, p*/, /t, t^h^, t*/, and /k, k^h^, k*/ received the lowest mean recognition scores of 18.6, 15.0, and 24.1% correct (Fig 3). Korean affricates /ʧ, ʧ^h^, ʧ*/ received a much higher mean recognition score (43.4% correct), however, they tended to be confused with the fricative /s/. Those Korean affricates are longer in duration and have a frication portion unlike stops that have only bursts and are very short.

In contrast to the three-way distinction of Korean stops, Korean fricatives /s/ and /s*/ form a two-way distinction [14]. The vocoder processed /s/ and /s*/ were recognized with the highest accuracy among all Korean consonants, 56.9% and 58.5% correct, respectively. However, they were often mutually confused (Fig 3). The voiceless glottal fricative /h/ was recognized only 25.2%, which may be related to the fact that the acoustic features of /h/ are unstable and easy to be affected by the adjacent sound. In addition, /h/ was confused with the voiceless /s/ (27.7% of the time) but not with the voiceless /s*/ (6.6% of the time) probably due to the tensing properties.

In terms of the last confusion group of /n/, /m/, and /l/, these three sounds are sonorants and have similar acoustic manifestation. In particular, the two nasal sounds showed high confusion rates with each other but not with /l/.

While Korean differed from English in the overall consonant-recognition performance and specific patterns as discussed above, the overall vowel-recognition performance of the two languages was similar. Like English listeners, Korean listeners also showed predominant reliance on the number of channels in vowel recognition (Fig 2). In addition, Korean listeners also showed a clear pattern of confusion, that is, higher confusion rates occurred within the groups of vowels that share the same nucleus vowels (Fig 4).

In sum, the present study demonstrated that the relative contributions of spectral and temporal cues to Korean phoneme recognition bore remarkable resemblance to that to English phoneme recognition under the vocoder processing. However, the vocoder processed Korean consonants showed markedly reduced overall recognition performance. The poor performance might be due to the more complex consonant repertoire of Korean, in particular, the three-way distinction of the stops. In addition, we also noticed certain asymmetries of stimuli-responses in both consonants and vowels (e.g., /pʰ/-/p*/ and /o/-/u/). This may be associated with the lexical frequency of the tokens containing the targets sounds. The present study used /Ca/ structure for consonant recognition which may not reflect the acoustic differences in closure duration because the stops were located at the initial position. Future studies should include /aCa/ structure that contains consonant in an inter-vocalic position. This would allow us to examine how the differences of closure duration in stops affect the recognition results. It is worth noting that vocoder processing tends to overestimate the speech-recognition performance of real cochlear implant users (e.g., [17, 18]). Therefore, it is imperative to verify our findings in Korean-speaking cochlear implant users. If results from the cochlear implant users are consistent with those obtained in the present study from the normal-hearing listeners listening to the vocoder-processed phonemes, current speech-processing strategies that are successful for Western languages should be modified to accommodate for Korean.

## Supporting Information

S1 Table Raw data for consonant recognition. Each Excel sheet contains data from one subject (N = 11). The first column represents talkers with 1 coded for the female and 2 coded for the male talker. Columns 2 and 3 are stimuli and responses with values from 1 to 18 representing ba, cha, da, ga, ha, ja, jja, ka, kka, ma, na, pa, ppa, ra, sa, ssa, ta, and tta, respectively. Column 4 presents the test conditions with number of channels and lowpass cutoff frequency labeled.
(XLS)

S2 Table Raw data for vowel recognition. Each Excel sheet contains data from one subject (N = 11). The first column represents talkers with 1 coded for the female and 2 coded for the male talker. Column 2 and 3 are stimuli and responses with values from 1 to 17 representing had, hid, heod, haed, heud, hod, hud, hyeod, hyad, hwad, hyod, hwod, hwid, hyaed, huid, hwaed, and hyud, respectively.
(XLS)

## References

[1] Dudley H. The vocoder. Bell Labs Rec. 1939; 17: 122–126.

[2] Shannon RV, Zeng F-G, Kamath V, Wygonski J, Ekelid M. Speech recognition with primarily temporal cues. Science. 1995; 270: 303–304. 7569981 10.1126/science.270.5234.303

[3] Dorman MF, Loizou PC, Rainey D. Speech intelligibility as a function of the number of channels of stimulation for signal processors using sine-wave and noise-band outputs. J Acoust Soc Am. 1997; 102: 2403–2411. 9348698 10.1121/1.419603

[4] Dorman MF, Loizou PC, Fitzke J, Tu Z. The recognition of sentences in noise by normal-hearing listeners using simulations of cochlear-implant signal processors with 6–20 channel. J Acoust Soc Am. 1998; 104: 3583–3585. 9857516 10.1121/1.423940

[5] Xu L, Thompson CS, Pfingst BE. Relative contributions of spectral and temporal cues for phoneme recognition. J Acoust Soc Am. 2005; 117: 3255–3267. 15957791 10.1121/1.1886405 PMC1414641

[6] Shannon RV, Fu Q-J, Galvin J. The number of spectral channels required for speech recognition depends on the difficulty of the listening situation. Acta Otolaryngol Suppl. 2004; 552: 50–54. 15219048 10.1080/03655230410017562

[7] Xu L, Tsai Y, Pfingst BE. Features of stimulation affecting tonal-speech perception: Implications for cochlear prostheses. J Acoust Soc Am. 2002; 112: 247–258. 12141350 10.1121/1.1487843 PMC1414789

[8] Kong YY, Zeng FG. Temporal and spectral cues in Mandarin tone recognition. J Acoust Soc Am. 2006; 120: 2830–2840. 17139741 10.1121/1.2346009

[9] Xu L, Zheng Y. Spectral and temporal cues for phoneme recognition in noise. J Acoust Soc Am. 2007; 122: 1758–1764. 17927435 10.1121/1.2767000

[10] Xu L, Pfingst BE. Spectral and temporal cues for speech recognition: Implications for auditory prostheses. Hear Res. 2008; 242: 132–140. doi: 10.1016/j.heares.2007.12.010 18249077 10.1016/j.heares.2007.12.010 PMC2610393

[11] Lee HB. Handbook of the International Phonetic Association. Cambridge, England: Cambridge University Press; 1999 pp. 120–122.

[12] Greenwood DD. A cochlear frequency-position function for several species—29 years later. J Acoust Soc Am. 1990; 87: 2592–2605. 2373794 10.1121/1.399052

[13] Warton DI, Hui FKC. The arcsine is asinine: the analysis of proportions in ecology. Ecology. 2011; 92: 3–10. 21560670 10.1890/10-0340.1

[14] Cho T, Jun S-A, Ladefoged P. Acoustic and aerodynamic correlates of Korean stops and fricatives. J Phonetics. 2002; 30: 193–228.

[15] Kim M-R, Duanmu S. “Tense” and “Lax” stops in Korean. J East Asian Ling. 2004; 13: 59–104.

[16] Han MS, Weitzman RS. Acoustic features of Korean /P, T, K/, /p, t, k/ and /ph, th, kh/. Phonetica. 1970; 22: 112–128.

[17] Svirsky MA, Ding N, Sagi E, Tan C-H, Fitzgerald M. Validation of acoustic models of auditory neural prostheses. ICASSP. 2013; 8629–8633. 10.1109/ICASSP.2013.6639350 PMC4244817 25435816

[18] Yang H-I, Zeng F-G. Reduced acoustic and electric integration in concurrent-vowel recognition. Sci Rep. 2013; 3: 1419 doi: 10.1038/srep01419 23474462 10.1038/srep01419 PMC3593224

